# Broca’s area network in language function: a pooling-data connectivity study

**DOI:** 10.3389/fpsyg.2015.00687

**Published:** 2015-05-22

**Authors:** Byron Bernal, Alfredo Ardila, Monica Rosselli

**Affiliations:** ^1^Brain Institute–Department of Radiology, fMRI and Neuroconnectivity, Miami Children’s HospitalMiami, FL, USA; ^2^Department of Communication Sciences and Disorders, Florida International UniversityMiami, FL, USA; ^3^Neuropsychology, Florida Atlantic UniversityDavie, FL, USA

**Keywords:** BA44, Broca, fMRI, connectivity, language, functional connectivity, ALE, MACM

## Abstract

**Background and Objective:** Modern neuroimaging developments have demonstrated that cognitive functions correlate with brain networks rather than specific areas. The purpose of this paper was to analyze the connectivity of Broca’s area based on language tasks.

**Methods**: A connectivity modeling study was performed by pooling data of Broca’s activation in language tasks. Fifty-seven papers that included 883 subjects in 84 experiments were analyzed. Analysis of Likelihood Estimates of pooled data was utilized to generate the map; thresholds at *p* < 0.01 were corrected for multiple comparisons and false discovery rate. Resulting images were co-registered into MNI standard space.

**Results:** A network consisting of 16 clusters of activation was obtained. Main clusters were located in the frontal operculum, left posterior temporal region, supplementary motor area, and the parietal lobe. Less common clusters were seen in the sub-cortical structures including the left thalamus, left putamen, secondary visual areas, and the right cerebellum.

**Conclusion**: Broca’s area-44-related networks involved in language processing were demonstrated utilizing a pooling-data connectivity study. Significance, interpretation, and limitations of the results are discussed.

## Introduction

One way to describe functional cortical organization is through maps that parcel the entire cortex into small regions, each one having specific brain functions. The most popular map was first described by Brodmann (1909) who subdivided the cortex of each hemisphere into 52 areas. These areas are segmented on the basis of histological differences, and do not have any concordance with the anatomical sub-divisions of the brain into lobes and gyri. Brodmann’s area 44 (BA44) is one of the few functional areas that has a precise correspondence with one of the anatomical subdivisions of the cortex. Indeed, BA44 is contained and limited by pars opercularis of the left inferior frontal gyrus, the core of the expressive language function described by Broca (1861).

Brodmann’s area 44 is involved in verbal fluency, phonological processing, grammar processing, attention in speech, sentence comprehension (e.g., Benson and Ardila, 1996; Kang et al., 1999; Grossman et al., 2002; Giraud et al., 2004; Sahin et al., 2006; Heim et al., 2008). In addition to these language functions, BA44 has been found active in many other non-verbal functions, including processing sequential sounds, working memory, mirror neuron systems, motor inhibition, object manipulation, and music enjoyment (Grossman et al., 2002; Giraud et al., 2004; Sahin et al., 2006; Zekveld et al., 2006).

It is troublesome to explain such a variety of functions from a segregationist model of brain function based on modules. Most modern models advocate multi-modular approaches explaining cognition as a variance in network configuration. This means that any specific area (e.g., BA44) may connect with different modules, depending of the task, yielding specific network configurations responsible for a given function. Brain connectivity, the term to refer to this view, may explain better complex cognitive, behavioral and neuropsychological phenomena than simple localization models. Neural network characterization models are of value in the present status of cognitive neuroscience, and to accomplish this quest input from different methods are necessary.

As a result, there has been newly found interest in brain connectivity by the advent of diffusion tensor imaging (DTI) and resting-state functional MRI (fMRI). DTI is capable of identifying fiber tracts of live neuronal tissue using a recently developed technique called tractography. Tractography is an imaging post-processing technique that merges minute water diffusion trajectories (tensors) in a chain that represents neural tracts. Resting-state fMRI is another form of computing post-processing procedure that depicts brain connections by representing discrete brain areas whose spontaneous oscillations are in synchrony. These two post-processing procedures are currently the most popular methods to study brain connectivity.

New terminology has been created to define the findings or technique associated with modern brain connectivity studies. (1) *Structural connectivity*, refers to the depiction of fibers by tractography; (2) *Functional connectivity*, refers to maps of synchronic brain oscillations, (3) *Effective connectivity*, refers to task-based fMRI in which statistical and heuristic approaches assess the direction of the data flow in the activated modules of fMRI. Tractography may be *deterministic* or *probabilistic*; functional connectivity may be based on Independent Component Analysis (ICA) of the whole data, or more limited depicting remote synchrony related to average of signal variation of a given region-of-interest (aka seeded-based functional connectivity). Effective connectivity may be explored with at least two quite complex mathematical approaches [*Structural Equation Modeling* and *Dynamic Causal Modeling* (DCM)].

Although all of these methods may provide similar results they may also differ. For example, the use of deterministic tractography has limited resolution with crossing fibers, while probabilistic tractography seems to trade sensitivity for specificity (Yo et al., 2009). Functional connectivity is observed between regions where there is little or no structural connection (Damoiseaux and Greicius, 2009). However, functional connectivity may change and can be influenced by rapid learning, training of a task specific performance or a lesion (Kelly and Garavan, 2005; Hasenkamp and Barsalou, 2012; Jolles et al., 2013; Vahdat et al., 2014) while structural connectivity is more stable and changes are mainly related to lesions. The vast majority of studies of functional connectivity are based on resting-state fMRI. Few studies have explored brain connectivity during or after tasks (e.g., Caclin and Fonlupt, 2006; Bernal et al., 2013; Dima et al., 2013). Brain activation maps in task-related fMRI have displayed connectivity but always limited to the function investigated. This is the substract of *effective connectivity*.

A recent described methodology based on a rather limited meta-analysis technique has also been described to depict brain functional networks. The method has been termed *meta-analytic connectivity model* (MACM; Bzdok et al., 2013; Ardila et al., 2014; Kohn et al., 2014). In the present study we utilize this method to describe Broca’s area core network involved in expressive language. We have preferred to term it “Pooling-data connectivity study” to avoid confusion with the standard meta-analysis methodology which usually requires broader sources.

## Materials and Methods

The data source for this pooling-data connectivity study was brainmap.org. The reason to utilize only this source is that this database provides specific and systematized fields of information and software-specific coding of activation coordinates that make possible precise, automatic and consistent selection of the sample to study. The output of the database is read also by a specific software, also provided by brainmap.org. Thus, the database of Brainmap^1^ was accessed utilizing Sleuth 2.2, open software provided by the same web site, on August 20th, 2014. The search conditions were: (1) studies reporting BA44 or Broca’s area activation; (2) studies using fMRI; (3) normal subjects; (4) activations: “activation only” (discarding report of de-activations); (5) right-handed subjects; (6) age 20–60 years; (7) domain: language. The search is automaticaly performed by Sleuth, giving a list of papers that satisfice the selecton criteria. Subjects over 60 were excluded because of two reasons, (1) Age is usually considered as a strong risk factor for cognitive decline in general, and dementia in particular (Ritchie and Kildea, 1995; Ferri et al., 2006); (2) some verbal ability decline is observed after the age of 60 (e.g., Alwin and McCammon, 2001; Ardila, 2007); certain verbal abilities, such verbal fluency –a language production ability involving BA44 – clearly declines after this age (Tombaugh et al., 1999).

Sixty-nine papers with 102 of 407 experiments matched criteria. Exclusion criteria were applied at this moment. Articles were excluded if they had bilingual subjects or had tasks performed in oriental languages, tasks limited to automatic speech, tasks limited to receptive language. Papers reporting patients or papers in which language involvement was marginal or ancillary of other cognitive tasks (memory, attention, inhibition) or mediated the subject’s responses were also excluded. Subjects of both sexes were included. Based on these exclusion criterias 12 articles were excluded. Thus, the fMRI results of 57 papers were pooled for further analysis providing 883 of 914 subjects, 84 of 338 experiments; 175 of 280 conditions; and 1247 of 3699 locations (**Table 1**). Activations associated to BA44 (search criteria) were obtained automatically from the Sleuth software. This automatic report list a number of clusters defined by the center of mass (in MNI coordinates), cluster volume in mm^3^, and intensity. These coordinates, per subject/task/paper were exported as text files (pooled resutls) for analysis on the following step.

**Table 1:** Activation likelihood estimate (ALE) report.

Cluster #	Anatomy. Brodmann’s area # (BA)	*x*	*y*	*z*	Vol mm^3^
1	L inferior frontal gyrus. BA44	-47.29	15.58	15.58	39992
	L anterior insula. BA13				
	L inferior frontal gyrus. BA9				
	L precentral gyrus. BA6				
	L inferior frontal gyrus. BA47				
	L middle frontal gyrus. BA46				
	L inferior frontal gyrus. BA46				
2	L superior frontal gyrus. BA6	-1.05	15.3	47.74	12904
	L medial frontal gyrus. BA32				
	R anterior cingulate gyrus. BA32				
3	L superior parietal lobule. BA7	-32.4	-56.45	46.44	12184
	L inferior parietal lobule. BA39				
	L supramarginal gyrus. BA40				
	L supramarginal gyrus. BA40				
	L superior parietal lobule. BA7				
4	R inferior frontal gyrus. BA44	42.59	19.39	1.71	10408
	R anterior insula. BA13				
	R insula. BA13				
	R inferior frontal gyrus. BA9				
	R putamen				
5	L fusiform gyrus. BA37	-43.49	-55.95	-16.62	5600
	L Cerebellum. Culmen.				
6	L middle temporal gyrus. BA22	-56.14	-43.5	7.37	4568
	L superior temporal gyrus. BA22				
	L middle temporal gyrus. BA22				
	L inferior parietal lobule. BA39				
7	L thalamus. Medial dorsal nucleus	-8.62	-13.64	9.57	1968
8	L lentiform nucleus. Putamen	-21.37	2.13	3.31	1728
	L lentiform nucleus. Putamen				
	L lentiform nucleus. Putamen				
9	Right superior parietal lobe. BA7	30.86	-57.4	46.16	1288
10	L inferior occipital gyrus. BA18	-28.72	-89.36	-1.72	1072
	L inferior occipital gyrus. BA19				
11	R middle occipital gyrus. BA18	33.36	-84.59	4.01	728
12	R fusiform gyrus. BA19	40.81	-72.77	-16.1	536
13	L cerebellum. Culmen.	-27.74	-61.62	-24.63	248
14	R precentral gyrus. BA4	52.64	-9.55	39.29	224
15	R cerebellum. Declive.	25.58	-67.76	-24.56	200
16	L superior temporal gyrus. BA22	-61.75	-15.11	6.26	200


Main loci of brain connectivity of Left BA44 (Broca’s Area) by meta-analytic connectivity modeling (MACM). Conventions: L, Left; R, Right; x, y, z: MNI coordinates; Vol, volumen of cluster in cubic millimiters as a measure of activation extent.

The statistical significance of clusters found on the pooled-data was then analyzed utilizing the activation likelihood estimate – (ALE) method. This step was performed with the open source software GingerALE^2^. ALE is a method to analyze coordinate-based brain activations in pooling-data studies. The description of the mathematics of ALE are beyond the purpose of this report. In a nutshell, ALE treats reported peaks of activation as spatial probability distributions centered at the given coordinates. ALE computes the union of activation probabilities for each voxel, allowing differentation between true convergence of activation foci from random clustering (noise). ALE scores obtained from thousands of random iterations are used to assign *p*-values to the observed clusters of activation. For more information on the theory of ALE the reader is advise to read the work of Eickhoff et al. (2009). Our ALE maps were threshold at *p* < 0.01 corrected for multiple comparisons with false discovery rate. Only clusters of 200 or more cubic mm where accepted as valid clusters. ALE results were overlaid onto an anatomical template suitable for MNI coordinates, also provided by brainmap.org. For this purpose we utilized the Multi-Image Analysis GUI (Mango)^3^. A mosaic of 3 × 3 transveral insets of fusion images was obtained utilizing the same tool, selecting every 3–4 images starting on image No. 10, and exported to a 2D-jpg image.

## Results

Sixteen significant clusters of activation were found with the ALE procedure. **Table 1** shows these clusters ranked by their volume in cubic millimeters. The main cluster encompasses BA44 and its abutting areas. These are the anterior insula, the inferior and middle frontal gyri and the pre-central gyrus. The second Cluster is located in the left pre-SMA and anterior cingulate gyrus involving BA6 and 32. The involvement of the right anterior cingulate gyrus could be real or, most likely, an effect of smoothing of the neighboring contralateral homologous structure. The third cluster is located in the left superior and inferior parietal lobule, an area shared by BAs 7, 39, and 40. The fourth cluster involved some mirror areas of the left Broca (right BA44, right anterior insula, and right BA9) and one subcortical structure, the putamen. The fifth cluster involved the left fusiform gyrus. The sixth cluster represents the core of the receptive language area or Wernicke’s area. The next cluster of importance was located in the left thalamus. Nine more clusters were listed in the automated report by GingerAle, as enumerated in **Table 1**, they are the left putamen, the right parietal lobe, the occipital lobes, the cerebellum, and the right precentral gyrus.

## Discussion

We present Broca’s area network specific to language tasks utilizing a method of ALE in pooled fMRI data. Our method differs from others by depicting the connectivity of a specific area in its widest range of potentiality, by focusing in the characterization of specific networks subserving a specific cognitive domain or function.

We found that language network of BA44 consists of 16 clusters. The first six clusters of activation are well established language areas: cluster 1 represents the left infero-lateral frontal gyrus and the anterior insula, that taken together have been recognized in the literature as the expressive language area (Benson and Ardila, 1996). The large size of the cluster reveals the dense neighboring connectivity to areas adjacent to BA44 through U fibers; cluster 2, represents the supplementary motor area, to which the prior is connected structurally through the aslant frontal fasciculus described by Catani et al. (2013), and most likely associated with verbal fluency and initiation of speech (Martino et al., 2012); cluster 3, represents the activation of the left superior and inferior parietal lobule, connected directly or indirectly with fibers of the arcuate fasciculus or the inferior occipitofrontal fasciculus (Dick et al., 2013). The connection to this parietal areas are more likely related with access to verbal working memory nodes (Jonides et al., 1998); cluster 4, showed activation of homologous areas of cluster 1; cluster 5 and 6 were related to canonical Wernicke’s areas, most likely connected through the arcuate fasciculus and subserving phonological transfering functions (Dick et al., 2013).

Clusters 7 to 16 consisted of activation of subcortical areas, medial dorsal nucleus of the left thalamus; left putamen and right cerebellum. The involvement of the left cerebellum is questionable and will be addressed later. Other small clusters are located in contralateral homologous areas of activations (BA7 and BA22), secondary visual areas (BA18 and BA19) in both hemispheres and activation of the right precentral gyrus. The involvement of these areas are not well understood. Visual areas may be involved in verbal tasks as the subject “re-visualize” objects and scenes described by the verbal material; precentral gyrus may be involved as a consecuence of subvocalization, that may be present when exposed to verbal material as a strategy to rehearsal imagery with motor clues (Smith et al., 1995). Of note is the lack of connectivity to left BA45 and left BA21, not listed in the ALE-automatic text report, nor appearing in the rendering image. The lack of involvement of left BA45 and BA39 will be addressed later.

To the best of our knowledge, no prior attempts to ascertain the *functional connectivity* of Broca’s area in language has been reported. Few publications, however, have reported studies assessing the brain connectivity related to specific tasks or language functions. Using a seed-based resting-state functional connectivity analysis Zhu et al. (2014) have demonstrated the language network seeding Broca’s and Wernicke’s areas. They demonstrated Broca’s to be left lateralized. Also, utilizing seed-based resting-state fMRI in a cohort of 970 healthy subjects Tomasi and Volkow (2012) found activation of the canonical prefrontal, temporal and parietal regions, bilateral caudate and left putamen/globus pallidus, and subthalamic nucleus. The authors utilized both Wernicke’s and Broca’s areas as seeding regions. There are also some studies of structural connectivity of Broca’s area. Morgan et al. (2009) reported combining DTI and resting-state functional connectivity to assess the connectivity between SMA and expressive language areas. In a meta-analytic study by Eickhoff et al. (2009) described the expressive network by pooling results of fluency tasks and conducting DCM (Heim et al., 2009). They found the core network consisting of BA44, anterior insula, BA6 (premotor cortex), and BA4 (primary motor cortex), with connections to basal ganglia and cerebellum. In their study they found that the DCM evidence the insula in a position between BA44 and two parallel nodes that include the cerebellum/basal ganglia and motor cortex. Heim et al. (2009) assessed the afferent connectivity to BA45 (and BA44, indirectly) in a language task of phonological/lexical discrimination visually presented.

Two additional studies have focused in parceling Broca’s area relating structure to function. The first study utilized probabilistic tractography and included BA45. Three segregated areas were identified: BA44, BA45, and deep opercular area abutting the anterior insula (Anwander et al., 2007); the second study utilized a method based on patterns of co-activation on several distinct cognitive tasks. In this work the authors describe five subdivisions of the Broca’s area, two posterior areas related to phonology (dorsal) and rhythmic sequencing (ventral), and three anterior areas related to working memory, switching control and semantics (Clos et al., 2013). It is not clear how this parcellation harmonizes with prior findings describing a dorso-ventral differentiation of BA44, allocating in the dorsal aspect the observation-related mirror neuron system (Molnar-Szakacs et al., 2005).

Our method although new is not completely novel. Sundermann and Pfleiderer (2012) conducted a study utilizing the same methodology as our study. These authors, however, did not target language. Instead, they focused their study in the “inferior frontal junction” (an area encompassing inferior frontal gyrus, caudal aspect of the middle frontal gyrus, and anterior insula), and its role in cognitive control. They applied meta-analytical connectivity modeling (MACM) based on the ALE method. As we did in our approach, they did their analysis aggregating all articles in which the left or right inferior frontal gyri were reported as activated. Like us they also made the area of activation the independent variable with no specific assumptions regarding functional specialization of the target area. All the co-activation areas were thought to be connected with the core area (selection criteria for the MACM).

The importance of characterizing the language network of BA44 lies in the aforementioned fact of multimodality involvement of this area across different domains in cognition. Broca’s area as the core of expressive language, seems to have other many functions. In addition to the functions already mentioned, BA44 appears involved in verbal working memory tasks (Rämä et al., 2001; Sun et al., 2005), particularly memory of syntactic type (Fiebach et al., 2005; Wang et al., 2008); mirror neuron system (Manthey et al., 2003; Lawrence et al., 2006; Lotze et al., 2006); motor programing (Amunts et al., 2004); tactile imagery (Yoo et al., 2003); arithmetic processing (Rickard et al., 2000); and even music enjoyment (Koelsch et al., 2006). This multifunctionality of Broca’s area may be explained in part by the anatomical subdivision described before, but still specific sub-areas should connect in a specific manner producing distinct task-related network configurations.

Broca’s sub-anatomical differentiation hypothesis is supported by a recent histological autoradiography study demonstrating different populations of cell receptors (Amunts et al., 2010). The main assumption in this work is that the differentiation in cell receptor segments also the function. There are six receptor types parceling Broca’s in a (1) ventral precentral transitional cortex, (2) dorsal BA44, (3) ventral BA44, (4) anterior BA45, (5) posterior BA45, and (6) middle frontal gyrus frontal operculum. Of note is the broader meaning of Broca’s area in this work, including BA45 or par triangularis of the IFG.

All these facts are indicative of a multi-potential function of BA44 that most likely has expression in a multiple-network configuration accounting for the different function output. Therefore a characterization of all the possible network configurations is advisable in an attempt to understand the plasticity of the brain function and the possible clinical effects of the local lesions.

Our results have important implications. It may serve as a point of departure to further caractherize the distinct Broca’s-related networks associated to diverse brain functions. It also may easy the explanation of the complexity of syndroms seen in speech and language disorders, difficult to reduce to two or three modules of the language standard model. The demonstration of specific networks subserving also specific cognitive functions, as presented in this paper, is important for cross validation of other techniques demonstrating brain connectivity; it also may serve to proof or dysproof the functional involvement of structural connectivity. For example, it has been found that some subjects with right hemisphere dominance for language, have left arcuate fasciculus dominance (Dick et al., 2013), in which case, the structural connectivity does not follows neural connectivity; our method may also evolve as a tool to acertain language lateralization if distinct right to left connectivities are demonstrated in future research on this field.

### Limitations

Many more articles have described BA44 activation in language tasks, but they have not been included in the brainmap.org database. To enter the database, study results have to report activation in standard space coordinates (MNI or Talairach), which have to be input manually to the database and then been approved by the team leading the brainmap.org project. Despite this limitation the authors estimate the number of studies/participants/experiments entering the pooling-data is large and reflects the state of the art publications in fMRI of language.

Another potential limitation derived from the pre-processing is the wrong allocation of activation in areas in which two different lobes or structures abut. Part of the preprocessing of the data consists of smoothing the activation. In smoothing, voxels with lower signal than the neighbors are increased for less noisy presentation. Thus, the algorithm treats all neighbors as a continuum. This procedure explains what is most likely a false activation of the culmen of the cerebellum, as the smoothing of the true activation obtained in the fusiform gyrus abuts the culmen. A similar effect explains the call of activation in the right anterior cingulate gyrus, and the left temporal superior gyrus (area 22 in cluster 1). This activation is most likely due to extension of the smoothing from the adjacent frontal operculum.

**FIGURE 1:**
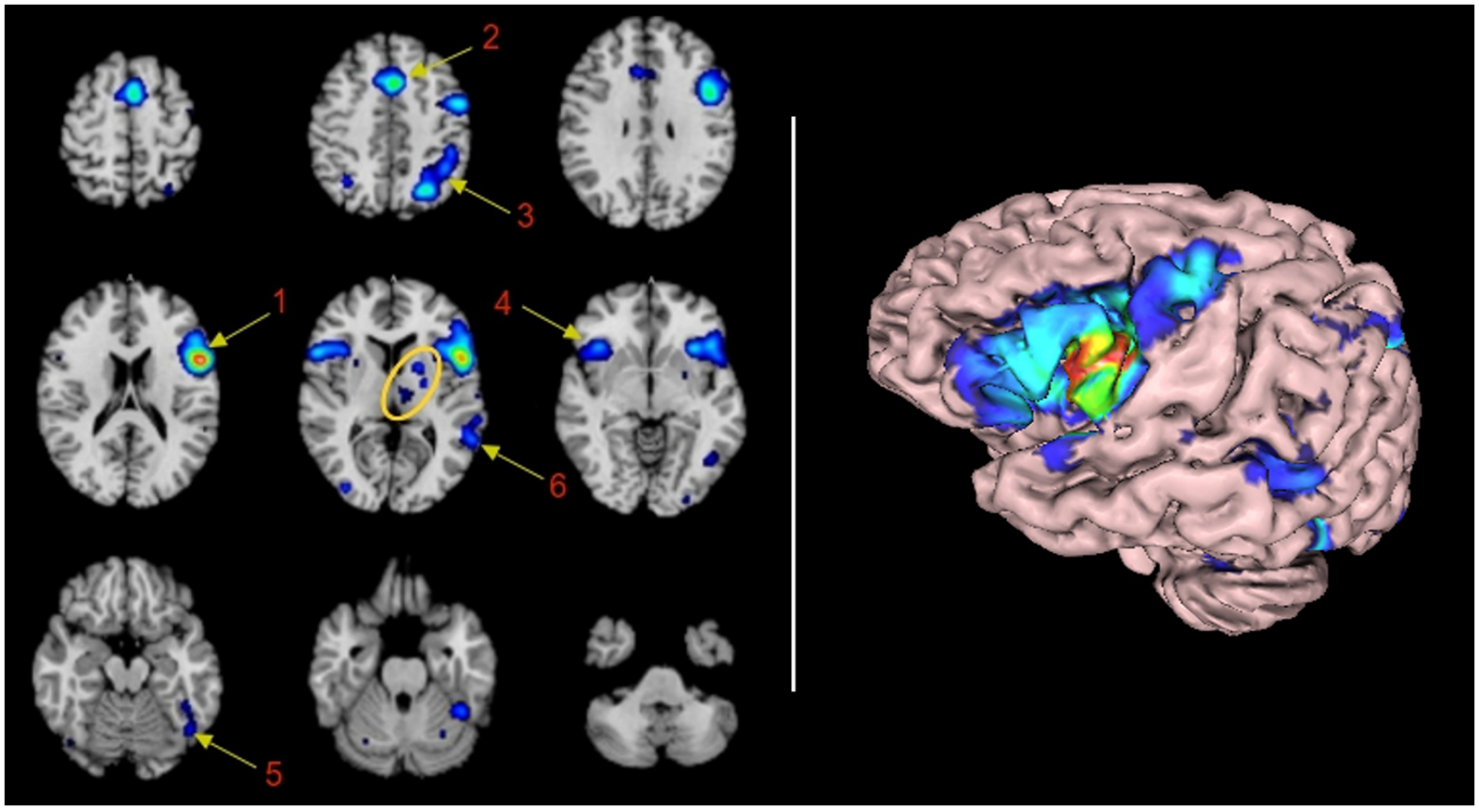
**Functional connectivity map of BA44 by Meta-analytic connectivity modeling. (Left)** Transversal descending cuts of the brain MRI template. Left hemisphere appears on the right side (Radiological convention). Clusters of activation are color coded for statistical significance from dark blue (lowest) to red (highest). Cluster numbers of the automatic activation likelihood estimate (ALE) report are associated with the main clusters of image. Arrows point approximately to their isocenters. Within the yellow oval, cluster 7 corresponds to the left thalamus, with medial localization, and cluster 8, lenticular nucleus, with lateral and rostral position. The cerebellar activation shown in the middle inset of the lower row is part of cluster 5. It is most likely explained by the smoothing effect of the adjacent activation of the left fusiform gyrus. **(Right)** 3D volumetric rendition of the brain showing activation on the left hemisphere surface. Red color zone identifies BA44. Deep and midline activations are not shown.

Two additional limitations should be exposed. Despite the clear involvement of BA45 in language demonstrated by fMRI studies aforementioned, this area is not mentioned in the ALE automatic report. Cluster 1 (**Table 1**) shows activation in all areas surrounding BA45 (i.e., 44, 47, 6, and 9). Therefore, it seems the algorithm is assuming a “block” including BA45 (Pars Tringularis) within BA44, since the activation is overt in that area according with the rendered image (**Figure 1**, right panel). A similar situation may explain the lack of report of BA21. These technological limitations are far from being suitable to be modified by the authors. However, *per se* they do not affect the statistical analysis or results.

Other limitations are conscious constrains of the study intended to avoid the effect of confounding factors that are known to affect language lateralization (righ-handed normal subjects, language type: occidental, and age: 20–60). The investigation of how these variables interplay with the Broca’s network is worth it to be tackle in future research.

The advent of a large database allows aggregation of information dependable under a given variable. It would allow us to obtain reliable information with high capability of generalization. In addition, pooling data in the method we propose here allows the demonstration of areas of co-activation across subjects and across tasks subserving either a specific function or a group of functions pertaining to a specific domain. In particular, each specific task (phonology, semantic, comprehension, etc) may use only a few of the modules. The depiction of high specific networks subserving specific functions may be of importance in clinical practice. For example, it could identify networks related to lateralization of language, and hence to help in neurosurgical planning in patients not suitable for task-dependent fMRI or Wada tests. At large, our method may demonstrate the possible or potential connectivity of the network for that cognitive domain enhancing the understanding of brain function. For example, we could assess all different “configurations” in which BA44 participates to reveal its maximum connectivity or, in a smaller scale, seek for the differentiation of such configurations to elucidate how BA44 is involved in various functions.

## Conclusion

We have demonstrated the application of a pooling data method to depict at large the network of BA44 related to language. The clusters of activation found are in line with prior clinical and neuroimaging studies, although the latter are scanty. For the sake of explaining brain function, the description of networks will have in the immidiate future a greater impact than the description of brain modules might have had in the past. Better comprehension of the brain connectivity will necessarilly help to better understand brain functions.

## Conflict of Interest Statement

The authors declare that the research was conducted in the absence of any commercial or financial relationships that could be construed as a potential conflict of interest.

## Footnotes

1 http://brainmap.org

2 http://brainmap.org

3 http://ric.uthscsa.edu/mango/
